# Consumption in Asylums

**Published:** 1899-11-04

**Authors:** 


### CONSUMPTION IN ASYLUMS.

A very striking paper by Dr. F. Graham Crookshank, in the *Journal of Mental Science*, draws attention afresh to a topic to which we have on many occasions referred, namely, the undue prevalence of phthisis among the insane confined in our public asylums. The more we know as to the causes of consumption, and every year our knowledge on the subject becomes more exact, the greater becomes the responsibility attaching to those who allow persons who are under their care to become tuberculous, especially when, as is the case with our asylum population, these persons are in regard to every detail of their lives absolutely under the control of those in whose charge they are placed. What seems clear is that the "official" death-rate from phthisis among our asylum population is four and a half times as high as the general phthisis death-rate for males between 35 and 45 years of age, that is, of the age group of the general population which is most liable to consumption. This of itself would betoken a serious enough condition of affairs, but there is much reason to believe that the "official" death-rate does not express the full gravity of the case. Owing to the obligation imposed upon those who compile the statistics of placing every death under some one heading, one can hardly doubt that in the case of certain diseases, as, for example, in the case of general paralysis of the insane, which is notoriously apt to end in consumption, the primary disease is often returned, to the exclusion of its tuberculous complication, and in this way the prevalence of phthisis, high as it is, is greatly understated in the official returns.

It has been the habit among some people to regard the insane as especially liable to contract tuberculosis, and it is not impossible that this may, to some extent, be the case, but it is a striking fact that only a small proportion of those who die of phthisis within the asylums show any signs of it on admission, or, indeed, for the first twelve months afterwards. At least four-fifths of these cases contract their disease while within the asylum walls. If there is one thing to which modern knowledge points more strongly than another, in regard to the conditions which conduce to consumption, it is that want of fresh air and the re-breathing of used air are at the bottom of the matter. Yet such are the very conditions to which many of the inmates of our asylums are condemned. "It would be difficult," we are told, "to find institutions which afford such opportunities for the dissemination of phthisis germs as do our asylums. Consider a community existing under conditions that preclude, for many, adequate exercise in the open air, spending long hours in overcrowded day-rooms and dormitories; a community of filthy and careless habits, and already phthisical in the proportion of from 15 to 25 per cent. Such a community is formed by the inmates of every county asylum. . . . Nowhere, save in asylums, are there such aggregations of tubercular persons whose malady is complicated by persistently uncleanly habits. . . . Small wonder then that farm labourers taken from the plough and placed in crowded wards die of phthisis in a year or two."

Dr. Crookshank has done well in drawing attention to this state of affairs, and especially in insisting that the three main causes at work—all of them preventable—are overcrowding in the asylums, lack of exercise in the fresh air, and a certain quality of diet. The overcrowding he proves by figures; in regard to the diet he says "no doubt their nutrition is poor, but it is childish to assert that half-a-crown or less per week is enough to spend on food." These things are things to rectify, but the first step of all should be to weed out those who are already consumptive, and place them in special asylums so that they may do no further harm to those who are still healthy. This, in the present state of our knowledge, is a perfectly elementary precaution, one which would occur to the merest tyro in sanitary science. Yet it is not done. Year after year we are building new asylums, bigger and bigger perhaps, but almost always on the old plan of accommodating all sorts of cases in each asylum. Surely it would be common sense to classify their inmates, and to plan certain of these new asylums for the reception of those only who are affected with consumption. This is not a matter of greater expense, but of the exercise of greater common sense, and a little more co-operation between different authorities in regard to the classification of the insane.

